# Investigation of Odor, Volatile Organic Compounds (VOC), and Total Organic Carbon (TOC) Parameters Originating from Textile Industry Stenter Stack

**DOI:** 10.3390/toxics14070560

**Published:** 2026-06-26

**Authors:** Ezgi Karabacak, Güray Çelik, Fatma Esen, Nezih Kamil Salihoğlu, Taner Yonar, Feza Örüç, Simge Çağlar, Berna Kırıl Mert

**Affiliations:** 1 Department of Environmental Engineering, Faculty of Engineering, Bursa Uludag University, 16059 Bursa, Türkiye; ezgikarabacak@hotmail.com (E.K.); gurays@uludag.edu.tr (G.Ç.); payan@uludag.edu.tr (F.E.); nkamils@uludag.edu.tr (N.K.S.); yonar@uludag.edu.tr (T.Y.); karaer@uludag.edu.tr (F.Ö.); simgecaglar22@gmail.com (S.Ç.); 2 Department of Environmental Engineering, Faculty of Engineering, Sakarya University, 54050 Sakarya, Türkiye

**Keywords:** textile industry, stenter stack, volatile organic compounds, odor, olfactometry

## Abstract

The textile industry causes significant environmental problems because of its intensive use of water, energy, and chemicals. The stenter machines, which are commonly used in textile finishing processes, release air pollutants such as odor, volatile organic compounds (VOCs), and total organic carbon (TOC) into the atmosphere during drying and fixing processes carried out at high temperatures. The aim of this study was to investigate odor, VOC, and TOC emissions from the stacks of the stenter machines. In this study, odor, VOC, and TOC parameters were examined in samples from the stacks of stenter machines of nine different plants operating in the textile sector in Bursa, Turkey. The samples were analysed in accordance with EN 13725:2022 standard, EN 13649:2014 standard, and EN 12619:2013 standard for odor, VOC, and TOC parameters, respectively. Acetone, carbon tetrachloride, dibromochloromethane, ethylbenzene, tetrachlorethylene, toluene, and p + m-Xylene were the most common components. The TOC concentrations were determined in the range of 13.89–279.23 mg/Nm^3^. The odor concentrations were determined in the range of 4113–26,627 OU/m^3^.

## 1. Introduction

The textile industry is indispensable for the basic human need of clothing and has a long supply chain that brings together many sub-sectors [1,2]. This chain includes processes such as raw material supply, finishing [3], dyeing [4], final product processing (clothing, home textiles, etc.), logistics [5], water management [6,7], and waste management [8,9]. Due to its long supply chain, textile industry activities result in numerous environmental problems. High energy and water consumption [10], wastewater production [8,9], solid waste production [11,12], air emissions [10,13], and odor [14] are the major environmental issues of the textile industry. Various chemicals and large quantities of water are used during textile manufacturing processes [15]. Water use generally occurs in two main stages: the application of chemicals to the textile substrate and the subsequent rinsing process [16]. As a result of the use of dyes, chemicals, auxiliary agents, and sizing materials during wet processing, the textile sector generates significant volumes of industrial wastewater [17]. Dyes, auxiliary chemicals, finishing agents, solvents, and various process chemicals used in textile manufacturing may lead to the generation of environmental emissions throughout different stages of the production process [18,19].

The textile industry uses energy intensively in its production processes. Heat consumption is particularly important in drying and curing processes, as well as in wet processing operations [20]. Approximately half of the total energy consumed in textile finishing plants is used for drying processes, and about 25% of this drying energy is attributed to stenter machines [21].

Several processes within textile manufacturing contribute to the generation of air emissions [22]. The most common air emissions in the textile industry include dust and lint, oil fumes, acid vapors, solvent mists, boiler stack gases, and odors [22].

The European Green Deal, published by the European Commission in 2019, aims for an extensive transformation across all sectors for the purpose of achieving climate neutrality by 2050 [23]. The textile sector is one of the priority sectors identified in the transformation period due to its significant environmental impacts [23]. Under the European Green Deal, the EU Strategy for Sustainable and Circular Textiles, prepared in line with the Circular Economy Action Plan, was published. This strategy aims to ensure that textile products are durable, fixable, reusable, and recyclable, to reduce the use of hazardous chemicals, and to reduce the environmental footprint [24]. The digital passport was proposed in the Circular Economy Action Plan published by the European Commission in 2020 as a tool to increase product traceability and sustainability [25]. The digital product passport was established under a legal framework through the Ecodesign for Sustainable Products Regulation (ESPR) [26]. It aims to provide traceability in a digital environment for basic information such as the manufacturer, content, repairability, reusability, and environmental impact of products [26]. These legal regulations accentuate the monitoring and management of process-related air pollutants for the textile industry.

A limited number of studies examined VOC types originating from polyester fabric production in the textile industry [13] and VOC types in the textile printing and dyeing industry [27]. Zhang (2020) [27] reported that the types of VOCs originating from the textile printing and dyeing process are ethyl acetate, toluene, 1-hexene, 2-methylpentane, n-hexane, 3-methylpentane, and methyl tert-butyl ether (MTBE). Qian (2022) [13] reported that the types of VOCs originating from polyester fabric production are isoprene, dichloromethane, methyl tert-butyl ether, n-hexane, 1,2-dichloroethane, ethylcyclohexane, toluene, p-xylene, n-decane, n-nonane, n-undecane, n-dodecane, acetone, acrolein, isopropanol, ethyl acetate, ethylene, acetic acid, acetaldehyde, p-xylene, castor oil polyoxyethylene ether, pentane, hexane, heptane, octane, butylated hydroxytoluene, tridecyl polyoxyethylene ether, hexylene glycol, and simethicone.

Although several studies have investigated air emissions from textile processes such as dyeing, printing, and polyester production, research focusing specifically on stenter stack emissions remains limited. Stenter machine stacks, which represent one of the primary sources of process-related air pollutant emissions in the textile industry, release key atmospheric pollutants such as odor, volatile organic compounds (VOCs), and total organic carbon (TOC) into the atmosphere. These emissions mainly occur during high-temperature drying and fixation processes due to the evaporation of finishing chemicals, softeners, and solvents. To date, no published study has systematically evaluated the relationship between source-specific VOC emissions, odor concentrations determined by dynamic olfactometry, and total organic carbon (TOC) levels in textile stenter stack exhaust, to the best of the authors’ knowledge. Furthermore, the adequacy of TOC as a proxy parameter for odor impact in stenter stack emissions has not been critically examined. This represents a significant gap, as emission control strategies and regulatory monitoring frameworks often rely on bulk parameters such as odor, VOC, or TOC without assessing their interrelation. Therefore, a detailed investigation of source-level emissions and the interrelationship between VOC, odor, and TOC is necessary to better characterize environmental impacts associated with textile industry operations.

Although odor, VOC, and TOC are frequently used as indicators of air emissions, they represent different aspects of the emission stream. VOC measurements provide information on volatile organic compounds present in the exhaust gas, whereas TOC reflects the overall organic carbon content. In contrast, odor concentration determined by dynamic olfactometry represents the sensory response to the entire emission mixture. Therefore, a direct relationship between these parameters cannot be assumed. Evaluating their interrelationship is important to determine whether commonly used regulatory indicators such as VOC or TOC can adequately represent odor impact and to improve the environmental assessment of textile stenter emissions.

Therefore, the aim of this study was to investigate odor, VOC, and TOC emissions from stenter machine stacks in nine textile plants and to evaluate the relationships among these parameters using statistical analyses in order to assess their environmental significance.

## 2. Materials and Methods

### 2.1. Sampling Collection

**Figure 1:**
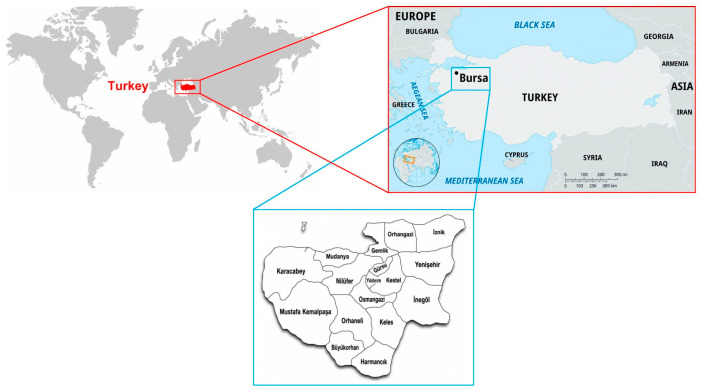
The map of sampling location.

**Figure 2:**
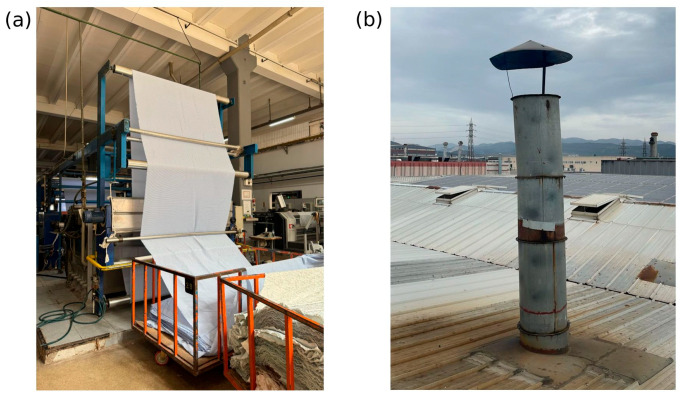
Stenter machine (**a**) and (**b**) stenter machine stack.

Figure 1 illustrates the study area in Bursa, Türkiye, where the sampling campaign was conducted. The study included nine textile plants operating within this region. Bursa was selected because it is one of the major textile production regions in Türkiye and includes a high number of textile finishing facilities using stenter machines. The selected plants represent real operating conditions of textile finishing processes in the region. Therefore, the results should be interpreted as source-level field data from the sampled plants rather than as sector-wide emission factors for the entire textile industry. The operational characteristics of the sampled textile plants are presented in Table 1. The samples were collected between April 2024 and March 2025 from stenter machine stacks. Figure 2 shows an example of a stenter machine and a stenter machine stack.

The stenter machines are used in textile finishing processes such as stretching, drying, heat setting, adjusting length and width, curing of chemical finishes [28,29]. The fabric is moistened or immersed in a chemical solution before the fabric is delivered to the stenter machine. The chemical solutions used ensure that the fabric possesses characteristics such as non-flammability and softness. The fabric is stretched and dried in a stenter machine at a temperature reaching 200 °C [28,29]. In this way, the fabric’s length and width are stabilized.

Stenter machines generate steam because they process textile products containing substances such as oil, plasticizers, and formaldehyde at high temperatures reaching up to 200 °C [14,28]. Stenter machine stacks ensure the release of vapour and volatile organic compounds, which are produced as a result of the high temperatures in the stenter machines, into the atmosphere. Stenter machine is one of the machines in the textile industry with the highest energy consumption due to high operating temperature and use of fans and burners [28,29,30]. Stenter machines are crucial for energy management and emission control because they are indispensable for fabric quality.

### 2.2. Method

In this study, all analyses were performed in triplicate for odor, VOC, and TOC. The relationships among odor, VOC, and TOC were examined using statistical methods with Pearson correlation, ANOVA, and *t*-test.

#### 2.2.1. Odor Analyses

**Figure 3:**
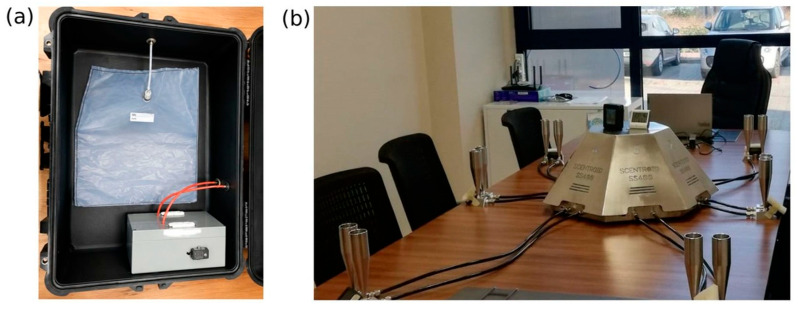
Sampling bag and vacuum chamber (**a**) and olfactometer (**b**).

The samples were collected from the stenter machine stack, which is a source of odor emission, using a polyethylene terephthalate (PET) sampling bag and a VC 20 Vacuum Chamber (Scentroid, Stouffville, ON, Canada) (Figure 3a) through the “Lung Method” [31]. The sampling bags were conditioned by filling and evacuating them with sample to approximately 10–20% of their total volume [31]. Samples were analysed in accordance with the EN 13725:2022 standard [31]. As shown in Figure 3b, odor analysis was carried out using a Scentroid SS400 mobile automatic dynamic dilution olfactometer (Scentroid, Stouffville, ON, Canada). The odor analyses were carried out by one panel leader and six panel members who had successfully completed the panel sensitivity test (expert nose training). The panel selection criterion was determined within the range of 20–80 ppb for panel members [31]. Training of panel members is required to ensure the reliability of the odor analysis results. Training details of panel members are provided in Supplementary Material Section S1 (Tables S1–S8). The odor-containing samples were diluted with neutral gas at various ratios and presented to the panel members within the olfactometer. Based on the answers of the panel members, the odor concentration of each sample was determined in odor units per cubic meter (OU/m^3^) [31].

#### 2.2.2. VOC Analyses

Before VOC analyses, the sampling of VOCs was performed by adsorbing VOCs onto activated carbon using a TCR TECORA DDS Dilution Sampling System (TCR TECORA, Milan, Italy). VOC Sampling was conducted at a flow rate of 0.5 L/min for 20 min, corresponding to a total sampling volume of 10 L for each sample. VOC analyses were performed using a Gas Chromatography–Mass Spectrometry (GC–MS) system, Agilent 5975B (Agilent, Santa Clara, CA, USA) in accordance with EN 13649:2014 standard [32]. The VOCs adsorbed on the activated carbon tubes were extracted with 1 mL of carbon disulfide (CS_2_). Sample extraction was performed by ultrasonic agitation for 10 min, followed by centrifugation for an additional 10 min prior to GC–MS analysis.

A 1 µL aliquot of the sample extract was injected into the GC. The concentrations of the VOCs were calculated by peak areas of the chromatograms. Extracts obtained from both the main adsorbent and the backup adsorbent layers were analysed separately. The GC–MS system was equipped with a capillary column (DB-VRX) with a film thickness of 1.4 µm, length of 60 m, and internal diameter of 0.25 mm. Mass spectral data were acquired over an *m*/*z* range of 10–1000. The total run time was approximately 40 min per sample, and the GC oven temperature was programmed from 40 °C to 225 °C. Helium was used as the carrier gas. Calibration solutions were prepared using the same extraction solvent used in the sample tubes. The concentration range covered the concentrations of the analysed sample extract. Calibration curves were generated using 2, 5, 20, 50, and 100 ppm working standards prepared from a certified CPA Chem 60 Components-2000 VOC standard solution (CPA Chem, Bogomilovo, Bulgaria). Calibration verification was performed using a CPA Chem 60 Components-200 standard solution (CPA Chem, Bogomilovo, Bulgaria).

#### 2.2.3. TOC Analyses

TOC analyses of gas samples collected from stenter machine stacks were carried out in accordance with EN 12619:2013 standard [33]. SIGNAL 3010 Minifid (Signal Group Ltd., Camberley, Surrey, Britain) device, which works on the flame ionization detector (FID) principle, was used to determine the carbon content of VOCs in the gas samples taken from the stenter machine stacks. The instrumental measurement range of the analyzer was 0–10,000 ppm.

The FID oven and catalyst temperatures were 190 ± 4 °C (adjustable between 130 and 210 °C) and 600 ± 10 °C, respectively. Heating time and response time were 30 min and 2 s, respectively. A deviation of up to ±1% could occur within a 10–30 °C ambient temperature variation. Instrumental stability was specified as a zero drift of ±0.2 ppm or ±2% of the measurement range (whichever is greater). The sampling flow rate was maintained between 0.4 and 2.5 L/min (±4%).

### 2.3. Quality Control (QC)/Quality Assurance (QA)

As part of the quality assurance and quality control (QA/QC) procedures, solvent blanks, laboratory blanks, and field blanks were analyzed. Carbon disulfide (CS_2_) was used as the solvent blank. In addition, parallel samples were collected to assess sampling and analytical repeatability. The data reliability was documented with measurement uncertainty for odor analysis. The detailed results and methodology of the measurement uncertainty assessment are provided in Supplementary Material Section S2 (Tables S9–S11). The accuracy of the method was verified using surrogate standards (CPA chem-60 components-2000/1028826 VOC Mixture, in Methanol, CPA Chem, Bogomilovo, Bulgaria) added prior to sample extraction for VOC analyses. Surrogate recovery rates were obtained above 80%. The correlation coefficients (R^2^) values were ensured above 0.99 for the calibration curves. For VOC analyses, the limit of detection (LOD) and limit of quantification (LOQ) were approximately 0.01 ppm and 0.03 ppm, respectively, while method detection limits (MDLs) were compound-dependent and within the same order of magnitude. The LOQ value was determined to be 1.55 ppm for TOC analyses.

## 3. Results and Discussions

### 3.1. Concentration and Species of VOCs Originating from Stenter Machine Stack

**Figure 4:**
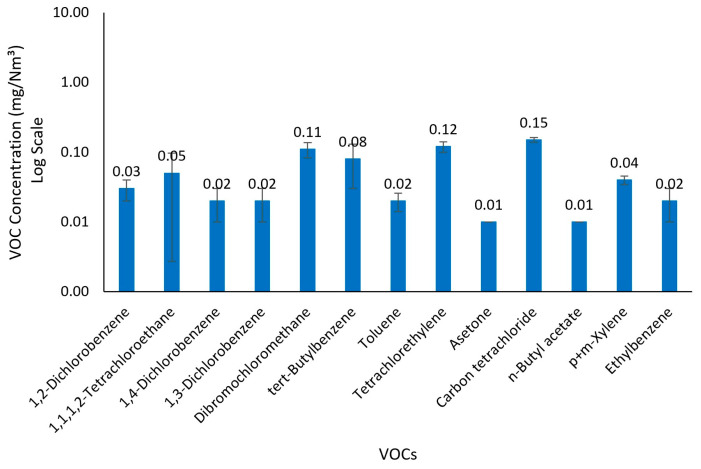
The species and concentration levels of VOCs originating from the stenter machine stacks of textile plant 4.

VOC species originating from the stenter machine stack of textile plant 4 and the average concentrations of these species are shown in Figure 4. VOC species and the average concentrations of these species are provided in Supplementary Material Section S3 (Figures S3–S10) for other plants. The species and frequency of occurrence of VOCs originating from the textile plants are shown in Supplementary Material Section S3 (Table S12). For nine textile plants, the total VOC concentrations were determined in the range of 0.49–10.97 mg/Nm^3^. For the Turkish Regulation, VOC limit levels are 20 mg/Nm^3^, 100 mg/Nm^3^, and 150 mg/Nm^3^ for Class I, Class II, and Class III, respectively [34]. The total VOC concentrations were below limit values for all nine textile plants.

The measured TVOC concentrations in the nine textile plants were 1.39, 2.14, 3.08, 0.69, 7.55, 1.63, 6.08, 10.97, and 0.49 mg/Nm^3^, respectively. According to the EU Textile BAT (the best available techniques) Conclusions, the BAT-AELs (Emission levels associated with the best available techniques) range for channelled TVOC emissions from textile processes including thermofixation or heat-setting is 3–40 mg/Nm^3^ [35]. None of the measured TVOC concentrations exceeded the upper BAT-AELs value of 40 mg/Nm^3^. TVOC concentrations measured in Plants 3, 5, 7, and 8 were within the BAT-AELs range, whereas those measured in Plants 1, 2, 4, 6, and 9 were below the lower end of the range. The BAT Conclusions indicate that the lower end of the BAT-AELs range is typically achieved when thermal oxidation is applied. Although stack gas control systems were present in the investigated plants, information regarding the use of thermal oxidation was not available. It should be noted that the lower end of the BAT-AELs range is typically associated with the application of thermal oxidation, and this should be considered when interpreting concentrations below 3 mg/Nm^3^ [35].

Although 50 target VOC species were included in the analytical scope of the study, as presented in Supplementary Material Section S3 (Table S12), only 35 VOC species were detected in the stack gas samples collected from textile stenter processes. The remaining target compounds were either not present in the analyzed samples or were below the instrumental detection limit. Therefore, they were not reported in the results section. Acetone, dibromochloromethane, carbon tetrachloride, ethylbenzene, tetrachlorethylene, toluene, and p + m-Xylene were detected in all nine textile plants. It is thought that these VOCs are typical VOCs for the textile finishing process. Additionally, n-butyl acetate was detected in 88.9% of the textile plants, while o-xylene and 2-propanol were detected in 77.8%. Some VOC species were detected in a limited number of textile plants. 1,2-Dichlorobenzene, 1,3-Dichlorobenzene, 1,4-Dichlorobenzene, tert-Butylbenzene, Methylene Chloride, and Isopropylbenzene were detected in 11% of the textile plants. On the other hand, n-Heptane, 1,2-Dibromo-3-chloropropane, and n-Butanol were detected in 22% of textile plants (Supplementary Material Section S3 Table S12). For a total of 50 possible VOC species examined, 20 different species were identified in plant 1 and 3, 18 species in plant 2, 14 species in plant 4, 26 species in plant 5, 22 species in plant 6, 19 species in plant 7, 17 species in plant 8, and 12 species in plant 9 (Supplementary Material Section S3 Figures S3–S10). It is thought that the differences between the plants may stem from variations in their production capacities, the types of raw materials, chemicals used, and processes. In Table 2, the species of VOCs originating from the textile industry are presented.

There are no extensive studies on the species of VOCs from stenter machine stack in the literature. For this reason, the VOC findings of this study were compared with general types of VOCs originating from the textile industry. Qian (2022) [13] reported that the types of VOC originating from polyester fabric production are toluene, p-xylene, acetone, isopropanol, and ethyl acetate. Zhang et al. (2020) [27] reported that the types of VOCs originating from the textile printing and dyeing process are ethyl acetate, toluene, and n-hexane. These VOCs were also frequently observed in this study. The species of VOC in this study’s stenter machine stack samples agree with the literature. However, some VOCs such as dibromochloromethane, 1,2-dibromo-3-chloropropane, sec- and tert-butylbenzene have not been reported in the literature and were observed only in this study. This situation is thought to be caused by differences in the textile production process, which result in the production of unique types of VOCs.

The observed differences in VOC, TOC, and odor concentrations among textile plants may be influenced by operational and process-related factors such as fabric type, chemical finishing agents, stenter operating temperature, residence time, ventilation configuration, exhaust gas dilution, capture efficiency, and the presence or absence of emission control systems. Information on fabric type, stenter operating temperature, emission control system type, and stack gas temperature was available for the investigated plants and is presented in the manuscript. However, other potentially influential operational parameters, including residence time, ventilation rate, exhaust gas flow, exhaust dilution, and capture efficiency, were not quantitatively measured or systematically recorded for all plants. Therefore, the influence of operational conditions on emission variability could not be comprehensively evaluated, and these factors should be interpreted as possible explanatory variables rather than statistically validated determinants of VOC, TOC, and odor concentrations. Elevated temperatures increase volatilization and can promote secondary formation of additional VOCs via thermal degradation of oils, binders, softeners, and polymer-related additives. This is consistent with the observation that stenter stacks can exhibit unique VOC profiles not commonly reported for other textile sub-processes.

The results of the stenter machine stack analysis show that odor formation in the textile industry is not limited to just a few known VOCs; on the contrary, this involves a fairly broad spectrum of compounds. Therefore, extensive monitoring and control strategies for VOCs should be developed for air quality and public health. Among the detected VOCs, benzene was classified by IARC as carcinogenic to humans (Group 1), while tetrachloroethylene and styrene were classified as probably carcinogenic to humans (Group 2A) [39]. Carbon tetrachloride, naphthalene, methylene chloride, and 1,2-dichloroethane have been classified as possibly carcinogenic to humans (Group 2B) [39]. These findings indicate that some of the detected VOCs may be of environmental and toxicological concern despite their relatively low concentrations. BTEX compounds, including benzene, toluene, ethylbenzene, and xylene derivatives, were detected in the stenter stack emissions. According to the literature, exposure to BTEX compounds may adversely affect multiple human physiological systems, including the respiratory, cardiovascular, digestive, urinary, hematological, hematopoietic, immune, reproductive, and nervous systems [40]. Therefore, the detected VOC profile highlights the environmental and potential health significance of textile stenter stack emissions in addition to their odor-related impacts.

### 3.2. Concentration of TOC Originating from Stenter Machine Stack

**Figure 5:**
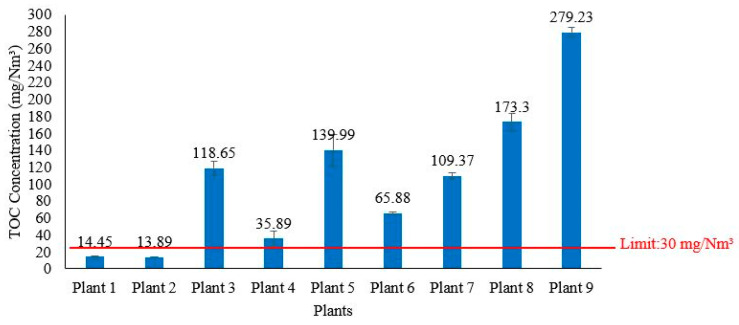
The concentration levels of TOC originating from the stenter machine stacks of textile plants.

**Figure 6:**
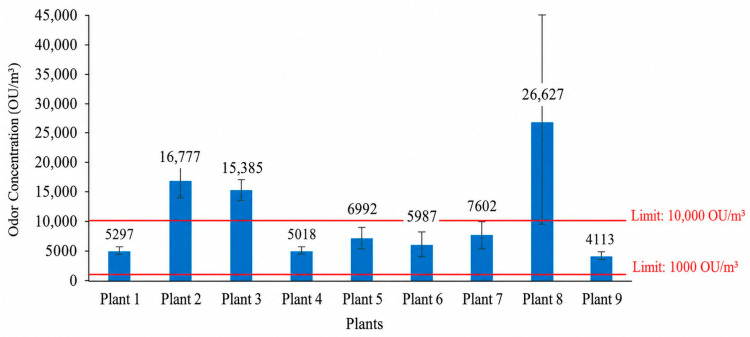
The concentration levels of odor originating from the stenter machine stacks of textile plants.

Figure 5 shows the variation in TOC concentration from the stenter machine stack of textile plants. The Turkish Regulation’s limit TOC value is 30 mg/Nm^3^ [34]. As shown in Figure 5, the TOC concentrations were determined in the range of 13.89–279.23 mg/Nm^3^. Except for plant 1 and plant 2 (Figure 5), TOC levels exceeded the legal limit value of Turkish Regulation. It was not possible to compare the TOC levels measured in this study with previously reported values, as TOC emissions from stenter machine stacks have not been reported in the literature. Concentrations of odor from the stenter machine stack of textile plants are shown in Figure 6.

### 3.3. Concentration of Odor Originating from Stenter Machine Stack

Odor concentrations measured in stenter machine stacks may vary among textile plants due to differences in ventilation systems, exhaust dilution, and emission capture efficiency. Consequently, even when emission rates are similar, dilution effects can lead to significant differences in the odor concentrations measured at the stack outlet.

According to the “Regulation on the Control of Odor Emissions” in force in Turkey, no measures are required for odor emissions below 1000 OU/m^3^ in odor measurements made at the source [41]. When odor levels are in the range of 1000–10,000 OU/m^3^, odor control measures must be taken at the plant [41]. In addition to this, administrative sanctions are imposed when odor level exceeds 10,000 OU/m^3^ [41]. As shown in Figure 6, levels of odor originating from the stenter machine stacks of all textile plants exceeded the limit of 1000 OU/m^3^ defined by the Turkish Regulation. The concentrations of odor from textile plants could not be compared with previous literature as no studies on this subject were published.

### 3.4. The Relationship Between Odor, VOC, and TOC

The correlation coefficients (Pearson correlations) of odor concentration, TOC, and VOC measured samples from the stenter machine stacks collected in textile plants are shown in Table 3. Each cell indicates the strength and direction of the relationship between two variables.

The correlation coefficient between odor and VOC was found to be r = 0.646, indicating a moderately strong positive correlation. As the VOC concentration increases, odor intensity tends to increase as well. The correlation coefficient between odor and TOC was found to be r = 0.072, suggesting a very weak correlation. The correlation coefficient between TOC and VOC was found to be r = 0.286, indicating a low-level positive correlation.

According to the correlation analysis, a significant positive relationship was observed between odor concentration and VOC concentration (r = 0.646). The results indicate that VOCs play a decisive role in odor formation.

On the other hand, a very weak correlation was observed between odor and TOC (r = 0.072), suggesting that the TOC parameter makes only a limited contribution to odor formation. This finding implies that the presence of organic carbon in the exhaust stream does not directly correspond to odor intensity. The correlation between TOC and VOC is low (r = 0.286). It is understood that these two parameters change partially together, but the relationship between them is not strong. The findings suggest that the monitoring and reduction of VOC emissions should be considered a key priority in odor management strategies for textile plants. Considering the effects of VOCs on both human health and the environment, controlling these compounds is important not only for odor management but also for improving overall air quality.

**ANOVA TEST**

The one-way ANOVA of odor, TOC, and VOC concentration measured in samples from the stenter machine stack of textile plants is presented in Table 4.

The ANOVA results (Table 4) showed that the mean square between groups (MS_between = 322,456,756.13) was substantially higher than the mean square within groups (MS_within = 19,263,791.89). According to the ANOVA result, the F statistic and *p*-value were calculated as 16.739 and 0.00003, respectively. Since this value is far below the significance level of α = 0.05, it indicates that there is a statistically significant difference among the groups (F(2,24) = 16.739; *p* < 0.001). The averages of odor, TOC, and VOC were determined as 10,421.8542 OU/m^3^, 105.6277778 mg/Nm^3^, and 3.78 mg/Nm^3^, respectively. ANOVA results showed that the measured values of odor, TOC, and VOC parameters differ significantly from one another. For this reason, it is necessary to consider separating these parameters in environmental monitoring, assessment, and control processes. The average and variance of odor values were much higher than those of VOC and TOC. This suggests that odor may be more strongly influenced by different processes or sources.

***t*-TEST**

The results of an independent samples *t*-test conducted to determine whether there is a statistically significant difference between odor and TOC parameters are shown in Table 5.

The *t*-test was conducted under the assumption that the variances of the two groups were unequal. The group variances differed considerably (odor: 57,783,955.79; TOC: 7406.76). The calculated t statistic was 4.071 with 16 degrees of freedom (df = 16). The obtained two-tailed *p*-value was 0.00089, while the critical *t* value was 2.1199. When evaluated at the 95% confidence level, these results indicate that there is a statistically significant difference between the mean values of odor and TOC (t(16) = 4.071; *p* < 0.001). The observed difference is too large to be attributed to random variation. Therefore, it is thought that odor values were significantly different from TOC values and these two parameters were affected by different environmental or process sources. This finding implies that, in identifying the causes of odor complaints, monitoring parameters more strongly correlated with odor (such as VOCs) may be more appropriate than relying solely on TOC measurements.

The results of an independent samples *t*-test conducted to determine whether there is a statistically significant difference between odor and VOC parameters are shown in Table 6.

The variances of the groups differed considerably (odor: 57,783,955.79; VOC: 13.14). Taking these differences into account, the calculated t statistic was 4.112 with 16 degrees of freedom (df = 16). The obtained two-tailed *p*-value was 0.00082, which is far below the critical *t* value of 2.1199. When evaluated at the 95% confidence level, these results indicate that there is a statistically significant difference between the mean values of odor and VOC (t(16) = 4.112; *p* < 0.001). The observed difference is too large to be attributed to random variation. Consequently, it was determined that odor emission levels are statistically and significantly higher than VOC levels. This finding suggests that odor formation is not solely attributed to VOCs, but may also be influenced by other volatile compounds or sulfur- and nitrogen-containing substances. In this context, odor control studies should consider not only VOCs but also other odor-active parameters in the assessment process.

The results of an independent samples *t*-test conducted to determine whether there is a statistically significant difference between total TOC and VOC parameters are shown in Table 7.

The variances were calculated as 7406.76 and 13.14, respectively. Based on these data, the t statistic was found to be 3.547 with 16 degrees of freedom (df = 16). The two-tailed *p*-value of 0.00268 was less than the critical *t* value of 2.1199. These data show that there is a statistically significant difference between the TOC and VOC averages (t(16) = 3.547; *p* < 0.01). This difference demonstrates that the variation in measurement results cannot be attributed solely to random fluctuations. The fact that TOC values are significantly higher than VOC values suggests that the measured organic carbon content is not limited to VOCs alone, but also includes contributions from other semi-volatile or gaseous organic compounds. This finding highlights the importance of jointly evaluating TOC and VOC measurements, particularly in the analysis of process emissions.

To further substantiate the relationship between odor and VOC concentrations and to address the predictive capacity of the measured parameters, a simple linear regression analysis was performed. While the Pearson correlation (r = 0.646) indicated a moderately strong positive association, the regression model provides a quantitative basis for predicting odor intensity based on VOC levels. The derived regression equation is

Odor (OU/m^3^) = 5298.29 + 1355.44 × VOC (mg/Nm^3^) (1)

The model yielded a coefficient of determination (R^2^) of 0.4176, indicating that approximately 42% of the variance in odor concentration can be explained by the VOC mass concentration alone. Despite the limited sample size (*n* = 9), the *p*-value (0.060) suggests a strong trend toward statistical significance, providing more robust support for the role of VOCs as a primary driver of odor emissions in stenter stacks compared to TOC (r = 0.072).

Furthermore, to quantify the precision of the experimental data, 95% confidence intervals (CI) were calculated for the primary emission parameters. The mean odor concentration was determined to be 10,421.85 OU/m^3^ with a CI of [4578.77, 16,264.94], while the VOC mean was 3.78 mg/Nm^3^ with a CI of [0.99, 6.57]. These intervals provide a transparent assessment of the measurement uncertainty and the inherent variability across the different textile facilities.

To evaluate the influencing factors on emission variability, a multiple regression approach was considered by incorporating operational temperatures. Analysis indicated that stack gas temperature serves as a contributing factor to the odor profile; for every 1 °C increase in stack gas temperature, the odor concentration showed a marginal upward trend (coefficient = 126.46), likely due to the enhanced volatilization of organic additives and the secondary formation of odor-active compounds at higher temperatures. While fabric type and emission control systems (e.g., electrostatic filters vs. wet scrubbers) contribute to the observed differences between plants, the VOC concentration remained the most significant quantitative predictor of odor impact in the investigated stenter processes.

## 4. Conclusions

This study provides an assessment of odor, VOC, and TOC emissions from stacks of stenter machines in textile plants. The results of this study show that stenter operations generate odor emissions.

A statistically significant positive relationship between VOC concentrations and odor levels in emissions from stenter machine stacks was observed. In contrast, the correlation between TOC and odor was weak, indicating that TOC measurements alone do not adequately reflect odor intensity in textile finishing emissions. The weak correlation observed between TOC and odor concentrations further demonstrates that TOC alone cannot adequately characterize odor intensity in textile stenter stack emissions. This finding suggests that odor perception is influenced not only by the total organic content of the emission stream but also by factors such as VOC composition, compound-specific odor thresholds, exhaust gas dilution, and the presence of odor-active substances. Therefore, TOC should be regarded as an indicator of overall organic emission load rather than a direct surrogate for odor impact.

The odor concentrations were determined in the range of 4113–26,627 OU/m^3^. The levels of odor originating from the stenter machine stacks of all textile plants exceeded the limit of 1000 OU/m^3^ defined by the Turkish Regulation.

The TOC concentrations were determined in the range of 13.89–279.23 mg/Nm^3^. The results of this study show that TOC, while relevant as a regulatory indicator of bulk organic loading, does not sufficiently represent odor impact.

Although TOC represents the overall carbon content, no information about the specific chemical composition of the emission stream can be inferred. On the other hand, odor levels depend strongly on the presence of individual compounds with low odor thresholds, even at relatively low concentrations. Therefore, TOC levels may fail to represent the odor-producing characteristics of complex emission mixtures such as those originating from stenter processes as observed in this study.

The VOC concentrations were determined in the range of 0.49–10.97 mg/Nm^3^. Acetone, carbon tetrachloride, Dibromochloromethane, Ethylbenzene, Tetrachlorethylene, Toluene, and p + m-Xylene were the most common VOCs. Some VOC species were detected in a limited number of textile plants. 1,2-Dichlorobenzene, 1,3-Dichlorobenzene, 1,4-Dichlorobenzene, tert-Butylbenzene, Methylene Chloride, and Isopropylbenzene were detected in 11% of the textile plants. On the other hand, n-Heptane, 1,2-Dibromo-3- chloropropane and n-Butanol were detected in 22% of textile plants. According to the correlation analysis, a significant positive relationship was observed between odor concentration and VOC concentration (r = 0.646). The odor relevance of the detected VOCs should be interpreted cautiously because odor formation depends not only on VOC concentration but also on compound-specific odor thresholds and mixture interactions. Therefore, the contribution of individual VOC species to odor formation could not be quantitatively evaluated within the scope of the present study. Future studies should include compound-specific odor characterization approaches to better evaluate the contribution of individual VOCs to odor formation in textile stenter stack emissions.

Air handling, exhaust dilution, and capture efficiency can be the reason for the different VOC and odor concentrations in textile plants. Differences in ventilation rates, hood design, and exhaust routing can alter the effective concentration measured at the stack. Even if mass emission rates were similar, stack concentrations can differ due to dilution, which affects both VOC and odor readings.

The plant-to-plant differences observed in this study may be associated with process and operational variability; however, these factors were not quantitatively validated and should be examined in future studies with detailed operational datasets. Elevated temperatures increase volatilization and can promote secondary formation of additional VOCs via thermal degradation of oils, binders, softeners, and polymer-related additives. This is consistent with the observation that stenter stacks can exhibit unique VOC profiles not commonly reported for other textile sub-processes.

Overall, the results suggest that monitoring strategies based solely on TOC limits may be insufficient for evaluating odor-related environmental impacts. VOC analysis combined with dynamic olfactometry can provide a more accurate environmental assessment of stack emissions.

The findings of this study provide field-based evidence on odor, VOC, and TOC emissions from stenter machine stacks in nine textile plants located in Bursa, Türkiye. However, since the study was conducted in a single region and did not employ a stratified sampling design, the results should be interpreted as representative of the investigated facilities rather than the entire textile industry. Although information on fabric type, stenter operating temperature, emission control system type, and stack gas temperature was available, other potentially influential operational parameters, including residence time, ventilation rate, exhaust gas flow rate, exhaust gas dilution, hood design, and capture efficiency, were not available as complete quantitative datasets. Consequently, the effects of operational conditions on VOC, TOC, and odor concentrations could not be comprehensively quantified. Future studies should include larger and more diverse facility datasets together with detailed operational monitoring to improve the representativeness of the findings and to identify the key factors influencing odor and VOC emissions from textile stenter operations.

## Figures and Tables

**Table 1:** Operational characteristics of the sampled textile plants.

Plant	Fabric Type	Stenter Operating Temperature	Emission Control System Type	Stack Gas Temperature
1	93% Viscose/7% Elastane	200 °C	Electrostatic filter	59.43 °C
2	89% Viscose/11% Polyamide	160 °C	Electrostatic filter	63.93 °C
3	90% Viscose/10% Elastane	160 °C	Wet scrubber	41.57 °C
4	99% Polyester/1% Elastane	160 °C	Electrostatic filter	48.69 °C
5	Polyester Bi-Stretch Fabric	190 °C	Wet scrubber	59.65 °C
6	50% Polyester/50% Cotton	160 °C	Electrostatic filter	57.19 °C
7	100% Polyester	190 °C	Wet scrubber	44.64 °C
8	80% Polyester/20% Elastane	190 °C	Electrostatic filter	57.05 °C
9	86% Polyamide 66/14% Elastane	160 °C	Electrostatic filter	56.70 °C

**Table 2:** The species of VOCs originating from the textile industry.

VOC Species	VOC Sources	Reference
Toluene, xylene, n-Hexane, n-Heptane, Isopropyl alcohol	Stenter stack	[36]
Ethanol, Methanol, Benzene, Methyl Acetate, Xylene, Cyclohexane, Trichloroethylene	Wastewater treatment plant	[37]
Butyl Acetate	Workplace ambient air	[38]
Ethyl Acetate, Toluene, 1-Hexene, 2-Methylpentane, n-Hexane, 3-Methylpentane, Methyl tert-butyl ether (MTBE)	Textile printing and dyeing	[27]
Isoprene, Dichloromethane, Methyl tert-butyl ether, n-Hexane, 1,2-Dichloroethane, Ethylcyclohexane, Toluene, p-Xylene, n-Decane, n-Nonane, n-Undecane, n-Dodecane, Acetone, Acrolein, Isopropanol, Ethyl Acetate, Ethylene, Acetic Acid, Acetaldehyde, p-Xylene, castor oil polyoxyethylene ether, Pentane, Hexane, Heptane, Octane, 2, 6-di-tert-butyl para-cresol, Tridecyl Polyoxyethylene Ether, Hexylene Glycol, Simethicone	Polyester fabric production in the textile industry	[13]

**Table 3:** Correlation matrix of odor, TOC, and VOC parameters in the stenter machine stack samples from textile plants.

	Odor (OU/m^3^)	TOC (mg/Nm^3^)	VOC (mg/Nm^3^)
Odor (OU/m^3^)	1		
TOC (mg/Nm^3^)	0.071636197	1	
VOC (mg/Nm^3^)	0.646253715	0.286037401	1

**Table 4:** ANOVA results of odor, TOC, and VOC parameters in the stenter machine stack samples from textile plants.

Source of Variance	SS	df	MS	F	*p*-Value	F Criterion
Between groups	6.449 × 10^8^	2	322,456,756.13203	16.73901	0.00003	3.40283
Within groups	4.623 × 10^8^	24	19,263,791.89			
Total	1.107 × 10^9^	26				

**Table 5:** Results of the independent samples *t*-test between odor and TOC.

	Odor (OU/m^3^)	TOC (mg/Nm^3^)
Mean	10,421.8542	105.6277778
Variance	57,783,955.79	7406.757069
Cumulative Variance	28,895,681.27	
Predicted Mean Difference	0	
df (Degrees of Freedom)	16	
t Stat	4.071089531	
P(T ≤ t) one-tailed	0.000444433	
t Critical one-tailed	1.745883676	
P(T ≤ t) two-tailed	0.000888866	
t Critical two-tailed	2.119905299	

**Table 6:** Results of the independent samples *t*-test between odor and VOC.

	Odor (OU/m^3^)	VOC (mg/Nm^3^)
Mean	10,421.8542	3.78
Variance	57,783,955.79	13.135675
Cumulative Variance	28,891,984.46	
Predicted Mean Difference	0	
df (Degrees of Freedom)	16	
t Stat	4.111544707	
P(T ≤ t) one-tailed	0.000408274	
t Critical one-tailed	1.745883676	
P(T ≤ t) two-tailed	0.000816548	
t Critical two-tailed	2.119905299	

**Table 7:** Results of the independent samples *t*-test between TOC and VOC.

	TOC (mg/Nm^3^)	VOC (mg/Nm^3^)
Mean	105.6277778	3.78
Variance	7406.757069	13.135675
Cumulative Variance	3709.946372	
Predicted Mean Difference	0	
df (Degrees of Freedom)	16	
t Stat	3.547104624	
P(T ≤ t) one-tailed	0.001341435	
t Critical one-tailed	1.745883676	
P(T ≤ t) two-tailed	0.002682871	
t Critical two-tailed	2.119905299	

## Data Availability

The original contributions presented in this study are included in the article/Supplementary Material. Further inquiries can be directed to the corresponding author.

## Supplementary Materials

The following supporting information can be downloaded at: https://www.mdpi.com/article/10.3390/toxics14070560/s1, Figure S1: Scentroid n-butanol sensitivity kit; Figure S2: Preparation of n-butanol sensitivity kit (a) transferring n-butanol to chamber (b) pressure adjustment (c) transferring n-butanol mixing to sampling bag (d) connecting the sampling bag to the olfactometry; Figure S3: The species and concentration levels of VOCs originating from the stenter machine stacks of textile plant 1; Figure S4: The species and concentration levels of VOCs originating from the stenter machine stacks of textile plant 2; Figure S5: The species and concentration levels of VOCs originating from the stenter machine stacks of textile plant 3; Figure S6: The species and concentration levels of VOCs originating from the stenter machine stacks of textile plant 5; Figure S7: The species and concentration levels of VOCs originating from the stenter machine stacks of textile plant 6, Figure S8: The species and concentration levels of VOCs originating from the stenter machine stacks of textile plant 7, Figure S9: The species and concentration levels of VOCs originating from the stenter machine stacks of textile plant 8, Figure S10: The species and concentration levels of VOCs originating from the stenter machine stacks of textile plant 9, Table S1: Expert nose training data for Panelist 1, Table S2: Expert nose training data for Panelist 2; Table S3: Expert nose training data for Panelist 3; Table S4: Expert nose training data for Panelist 4; Table S5: Expert nose training data for Panelist 5; Table S6: Expert nose training data for Panelist 6; Table S7: Expert nose training data for Panel Leader; Table S8: The data of the expert nose training and criteria; Table S9: Paired odor concentration measurements of environmental samples; Table S10: Odor concentration measurements of the certified reference materials, Table S11: Corrected paired odor concentrations of the environmental samples; Table S12: VOC species at textile plants.

## Abbreviations

The following abbreviations are used in this manuscript:

BAT	The best available techniques
BAT-AELs	Emission levels associated with the best available techniques
BTEX	Benzene, ethylbenzene, toluene, and xylene
VOC	Volatile organic compounds
TOC	Total organic carbon

## References

[1] Farhana K., Kadirgama K., Mahamude A.S.F., Mica M.T. (2022). Energy Consumption, Environmental Impact, and Implementation of Renewable Energy Resources in Global Textile Industries: An Overview towards Circularity and Sustainability. Mater. Circ. Econ..

[2] Rovira J., Domingo J.L. (2019). Human Health Risks Due to Exposure to Inorganic and Organic Chemicals from Textiles: A Review. Environ. Res..

[3] Gabriela K., Jiří M. (2025). Changes in Hairiness of Woven Fabrics at the Production and Finishing Stages. Sci. Rep..

[4] Chen Z., Liu J., Li J., Yuan M., Yu G. (2024). Leveraging Multi-Output Modelling for CIELAB Using Colour Difference Formula towards Sustainable Textile Dyeing. Auton. Intell. Syst..

[5] Ara M.A., Tamanna M.S., Ali M., Amin A., Shabur M.A. (2025). A Hybrid AHP and Statistical Validation Approach for Sustainable Supplier Selection in Apparel Industry. Discov. Sustain..

[6] Hussein A., Scholz M. (2018). Treatment of Artificial Wastewater Containing Two Azo Textile Dyes by Vertical-Flow Constructed Wetlands. Environ. Sci. Pollut. Res..

[7] Yaseen D.A., Scholz M. (2018). Treatment of Synthetic Textile Wastewater Containing Dye Mixtures with Microcosms. Environ. Sci. Pollut. Res..

[8] Khan M.I., Islam M.T., Wang L., Padhye R. (2025). Comparative Energy Demand and Carbon Footprint Analysis of Textile Waste Management Systems in Australia. Environ. Sci. Pollut. Res..

[9] Leenders N., Moerbeek R.M., Puijk M.J., Bronkhorst R.J.A., Bueno Morón J., van Klink G.P.M., Gruter G.-J.M. (2025). Polycotton Waste Textile Recycling by Sequential Hydrolysis and Glycolysis. Nat. Commun..

[10] Hasanbeigi A., Price L. (2015). A Technical Review of Emerging Technologies for Energy and Water Efficiency and Pollution Reduction in the Textile Industry. J. Clean. Prod..

[11] Stanescu M.D. (2021). State of the Art of Post-Consumer Textile Waste Upcycling to Reach the Zero Waste Milestone. Environ. Sci. Pollut. Res..

[12] Mishra P.K., Izrayeel A.M.D., Mahur B.K., Ahuja A., Rastogi V.K. (2022). A Comprehensive Review on Textile Waste Valorization Techniques and Their Applications. Environ. Sci. Pollut. Res..

[13] Qian W., Guo Y., Wang X., Qiu X., Ji X., Wang L., Li Y. (2022). Quantification and Assessment of Chemical Footprint of VOCs in Polyester Fabric Production. J. Clean. Prod..

[14] Toprak T., Anis P. (2017). Textile Industry’s Environmental Effects and Approaching Cleaner Production and Sustainability: An Overview. J. Text. Eng. Fash. Technol..

[15] Ghaly A.E., Ananthashankar R., Alhattap M.T., Ramakrishnan V.V. (2013). Production, Characterization and Treatment of Textile Effluents: A Critical Review. J. Chem. Eng. Process Technol..

[16] Ntuli F., Ikhu-Omoregbe D., Kuipa P.K., Muzenda E., Belaid M. Characterization of Effluent from Textile Wet Finishing Operations. Proceedings of the World Congress on Engineering and Computer Science (WCECS).

[17] Madhav S., Ahamad A., Singh P., Mishra P.K. (2018). A Review of Textile Industry: Wet Processing, Environmental Impacts, and Effluent Treatment Methods. Environ. Qual. Manag..

[18] Hasanuzzaman, Bhar C. (2016). Indian Textile Industry and Its Impact on the Environment and Health. Int. J. Inf. Syst. Serv. Sect..

[19] Kant R. (2012). Textile Dyeing Industry an Environmental Hazard. Nat. Sci..

[20] International Finance Corporation (IFC), World Bank Group (2007). Environmental, Health, and Safety Guidelines for Textile Manufacturing.

[21] Beckwith W.F., Beard J.N. (1979). A Scheme to Assist in the Evaluation of Tenter Frame Dryer Performance. J. Eng. Ind..

[22] Islam M.T., Islam T., Islam T., Repon M.R. (2022). Synthetic Dyes for Textile Colouration: Process, Factors and Environmental Impact. Text. Leather Rev..

[23] European Commission (2019). The European Green Deal. Communication from the Commission to the European Parliament, the European Council, the Council, the European Economic and Social Committee and the Committee of the Regions.

[24] European Commission (2022). EU Strategy for Sustainable and Circular Textiles. Communication from the Commission to the European Parliament, Communication from the Commission to the European Parliament, the Council, the European Economic and Social Committee and the Committee of the Regions.

[25] European Commission (2020). A New Circular Economy Action Plan for a Cleaner and More Competitive Europe. Communication from the Commission to the European Parliament, the Council, the European Economic and Social Committee and the Committee of the Regions.

[26] European Commission (2024). Regulation (EU) 2024/1781 of the European Parliament and of the Council of 13 June 2024. Establishing a framework for the setting of ecodesign requirements for sustainable products, amending Directive (EU) 2020/1828 and Regulation (EU) 2023/1542 and repealing Directive 2009/125/EC.

[27] Zhang Y., Li C., Yan Q., Han S., Zhao Q., Yang L., Liu Y., Zhang R. (2020). Typical Industrial Sector-Based Volatile Organic Compounds Source Profiles and Ozone Formation Potentials in Zhengzhou, China. Atmos. Pollut. Res..

[28] Alam S.M.M., Kibria M.G. (2022). Sustainable Heat-Setting Process in Stenter for Textile Industry. Mater. Today Proc..

[29] Choudhury A.K.R. (2017). Principles of Textile Finishing.

[30] Abdel-Dayem A.M., Mohamad M.A. (2001). Potential of Solar Energy Utilization in the Textile Industry—A Case Study. Renew. Energy.

[31] (2022). Stationary Source Emissions—Determination of Odour Concentration by Dynamic Olfactometry and Odour Emission Rate.

[32] (2014). Stationary Source Emissions—Determination of the Mass Concentration of Individual Gaseous Organic Compounds—Sorptive Sampling Method Followed by Solvent Extraction or Thermal Desorption.

[33] (2013). Stationary Source Emissions-Determination of the Mass Concentration of Total Gaseous Organic Carbon-Continuous Flame Ionisation Detector Method.

[34] Republic of Türkiye Ministry of Environment, Urbanization and Climate Change Regulation on Control of Industrial Air Pollution. Official Gazette No. 27277, 3 July 2009.

[35] European Commission (2022). Commission Implementing Decision (EU) 2022/2508 of 9 December 2022 Establishing the Best Available Techniques (BAT) Conclusions, under Directive 2010/75/EU of the European Parliament and of the Council on Industrial Emissions, for the Textiles Industry. https://eur-lex.europa.eu/eli/dec_impl/2022/2508/oj/eng/.

[36] Tahiroglu Y. (2019). Investigation of Sources and Control Methods of Volatile Organic Compounds Emitted from Textile Stenter Stacks. Master’s Thesis.

[37] Yang Z., Li J., Liu J., Cao J., Sheng D., Cai T. (2019). Evaluation of a Pilot-Scale Bio-Trickling Filter as a VOCs Control Technology for the Chemical Fibre Wastewater Treatment Plant. J. Environ. Manag..

[38] Müezzinoglu A. (1998). Air Pollutant Emission Potentials of Cotton Textile Manufacturing Industry. J. Clean. Prod..

[39] IARC Monographs on the Identification of Carcinogenic Hazards to Humans—List of Classifications. https://monographs.iarc.who.int/list-of-classifications/.

[40] Saeedi M., Malekmohammadi B., Tajalli S. (2024). Interaction of Benzene, Toluene, Ethylbenzene, and Xylene with Human’s Body: Insights into Characteristics, Sources and Health Risks. J. Hazard. Mater. Adv..

[41] Republic of Türkiye Ministry of Environment, Urbanization and Climate Change Regulation on Control of Emissions that Contribute to Odor. Official Gazette No. 28712, 19 July 2013.

