# Supplementary material for: Investigation of Odor, Volatile Organic Compounds (VOC), and Total Organic Carbon (TOC) Parameters Originating from Textile Industry Stenter Stack
Source: Toxics. 2026 Jun 26;14(7):560. doi: 10.3390/toxics14070560 (PMC13417753; doi:10.3390/toxics14070560)
Supplement: Supplementary file 1 [file toxics-14-00560-s001.zip › toxics-4332470-supplementary.pdf]

Supplementary Material

Investigation Of Odor, Volatile Organic Compounds (VOC), and Total Organic Carbon (TOC) Parameters Originating from Textile Industry Stenter Stack

1.SUPPLEMENTARY MATERIAL – Expert Nose Training

As part of expert nose training, n-butanol, a reference material known for its concentration, is presented to panelists at different dilution ratios. The n-butanol at different dilution ratios were analyzed by the olfactometry. Expert nose training was conducted following EN 13725:2022 Stationary source emissions-Determination of odor concentration by dynamic olfactometry and odor emission rate (EN 13725 2022).

The n-butanol sensitivity kit and preparation steps are shown in Fig. S 1 and Fig. S 2, respectively. The n-butanol sensitivity kit was used to prepare n-butanol solutions with concentrations ranging from 4 to 40 ppm. Firstly, 1 µl n-butanol was transferred into the chamber using a syringe. The pressure was adjusted to 5.5 bar. After waiting for 45 minute, n-butanol mixing in chamber was transferred to sampling bag. Finally, the sampling bag was connected to the olfactometry and n-butanol mixing was analyzed with olfactometry.

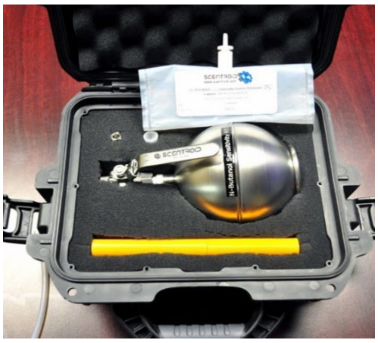

Fig. S 1. Scentroid n-butanol sensitivity kit

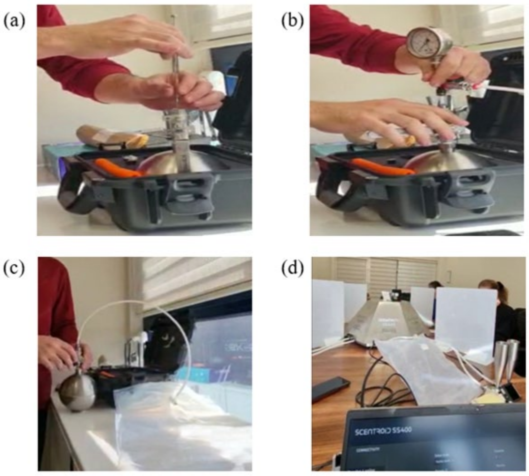

Fig. S 2. Preparation of n-butanol sensitivity kit a) transferring n-butanol to chamber; b) pressure adjustment; c) transferring n-butanol mixing to sampling bag; d) connecting the sampling bag to the olfactometry.

For each panelist, 12 individual odor threshold measurements were performed. Odor concentrations (OU/m^3) were converted to reference concentrations ($\mu mol\ mol^{-1}$) and logarithmically transformed (EN 13725 2022). The expert nose training values for each panelist and panel leader are shown in Table S1, Table S2, Table S3, Table S4, Table S5, Table S6, and Table S7. The assessment of the expert nose training results for each panelist and panel leader according to the criteria is shown in Table S8. The panelists' $\bar{Y}_{ITE}$ (average of individual threshold estimate) and S_{ITE} (standard deviation of individual threshold estimate) results are provided in Table S8. Table S8 also includes the criteria for panelists according to EN 13725. According to expert nose training results (Table S8), all panelists and the panel leader met the criteria specified in EN 13725.

Table S1. Expert nose training data for Panelist 1

y1	y2	y3	y4	y5	y6	y7	y8	y9	y10	y11	y12	Unit
1448	1448	724	1448	1448	1448	1448	724	724	362	1448	1448	OU/m^3
0.0276	0.0276	0.0552	0.0276	0.0276	0.0276	0.0276	0.0552	0.0552	0.1105	0.0276	0.0276	µmol/mol
−1.559	−1.559	−1.258	−1.559	−1.559	−1.559	−1.559	−1.258	−1.258	−0.957	−1.559	−1.559	log_{10} (µmol/mol)

Table S2. Expert nose training data for Panelist 2

y1	y2	y3	y4	y5	y6	y7	y8	y9	y10	y11	y12	Unit
1448	1448	2896	1448	1448	362	1448	1448	2896	1448	2896	2896	OU/m^3
0.0276	0.0276	0.0138	0.0276	0.0276	0.1105	0.0276	0.0276	0.0138	0.0276	0.0138	0.0138	µmol/mol
−1.559	−1.559	−1.860	−1.559	−1.559	−0.957	−1.559	−1.559	−1.860	−1.559	−1.860	−1.860	log10 (µmol/mol)

Table S3. Expert nose training data for Panelist 3

y1	y2	y3	y4	y5	y6	y7	y8	y9	y10	y11	y12	Unit
2896	1448	1448	2896	5793	724	724	724	1448	1448	724	1448	OU/m^3
0.0138	0.0276	0.0276	0.0138	0.0069	0.0552	0.0552	0.0552	0.0276	0.0276	0.0552	0.0276	µmol/mol
−1.860	−1.559	−1.559	−1.860	−2.161	−1.258	−1.258	−1.258	−1.559	−1.559	−1.258	−1.559	log_{10} (µmol/mol)

Table S4. Expert nose training data for Panelist 4

y1	y2	y3	y4	y5	y6	y7	y8	y9	y10	y11	y12	Unit
1448	724	1448	724	1448	2896	1448	724	1448	724	1448	1448	OU/m^3
0.0276	0.0552	0.0276	0.0552	0.0276	0.0138	0.0276	0.0552	0.0276	0.0552	0.0276	0.0276	µmol/mol
−1.559	−1.258	−1.559	−1.258	−1.559	−1.860	−1.559	−1.258	−1.559	−1.258	−1.559	−1.559	log_{10} (µmol/mol)

Table S5. Expert nose training data for Panelist 5

y1	y2	y3	y4	y5	y6	y7	y8	y9	y10	y11	y12	Unit
2896	2896	1448	1448	724	1448	724	1448	1448	724	2896	1448	OU/m^3
0.0138	0.0138	0.0276	0.0276	0.0552	0.0276	0.0552	0.0276	0.0276	0.0552	0.0138	0.0276	µmol/mol
−1.860	−1.860	−1.559	−1.559	−1.258	−1.559	−1.258	−1.559	−1.559	−1.258	−1.860	−1.559	log_{10} (µmol/mol)

Table S6. Expert nose training data for Panelist 6

y1	y2	y3	y4	y5	y6	y7	y8	y9	y10	y11	y12	Unit
1448	1448	724	724	2896	2896.3	2896.3	2896	1448	2896	1448	2896	OU/m^3
0.0276	0.0276	0.0552	0.0552	0.0138	0.0138	0.0138	0.0138	0.0276	0.0138	0.0276	0.0138	µmol/mol
-1.559	-1.559	-1.258	-1.258	-1.860	-1.860	-1.860	-1.860	-1.559	-1.860	-1.559	-1.860	log_{10} (µmol/mol)

Table S7. Expert nose training data for Panel Leader

y1	y2	y3	y4	y5	y6	y7	y8	y9	y10	y11	y12	Unit
724	2896	1448	1448	1448	2896	2896	2896	724	1448	2896	2896	OU/m^3
0.0552	0.0138	0.0276	0.0276	0.0276	0.0138	0.0138	0.0138	0.0552	0.0276	0.0138	0.0138	µmol/mol
-1.258	-1.860	-1.559	-1.559	-1.559	-1.860	-1.860	-1,860	-1.258	-1.559	-1.860	-1.860	log_{10} (µmol/mol)

Table S8. The data of the expert nose training and criteria

Parameter	Panelist 1	Panelist 2	Panelist 3	Panelist 4	Panelist 5	Panelist 6	Panel Leader	Limit (min)	Limit (max)
$\bar{y}_{ITE}$	−1.4333	−1.6089	−1.5587	−1.4082	−1.6841	-1.6591	-1.6591	-	-
s_{ITE}	0.2013	0.2513	0.2870	0.2401	0.2013	0.2344	0.2344	-	-
$10^{s_{ITE}}$	1.59	1.78	1.94	1.74	1.59	1.72	1.72	-	2.30
$10^{\bar{y}_{ITE}}$	0.037	0.025	0.028	0.039	0.021	0.022	0.022	0.020	0.080

2. SUPPLEMENTARY MATERIAL – Measurement Uncertainty Assessment

The measurement uncertainty assessment was conducted with panelists who participated in expert nose training and met the conditions (EN 13725 2022). The uncertainty resources are listed below as follows:

- Reference Material (n-butanol): Measurements were performed using certified reference material to test compliance with the quality criteria regarding the accuracy of the measurements.
- Panel member (temporal variation): Measurements were performed over three non-consecutive days to assess the individual variability and sensitivity of panelists. In total, 40 µmol/mol n-butanol was used for measurement.
- Panel composition (difference between panelists): For determining the differences between panelists, measurements were performed with n-butanol and samples collected from textile plants

The analysis results of samples collected from stenter stacks of textile plants are shown in Table S9. The analysis results of n-butanol are shown in Table S10. Corrected paired odor concentrations of the environmental samples are shown in Table S11.

Table S9. Paired odor concentration measurements of environmental samples

Date	$c_{od,a}$ ou_E/m^3	$c_{od,b}$ ou_E/m^3	$\log_{10}(c_{od,a})$	$\log_{10}(c_{od,b})$	$\overline{\log_{10}(c_{od})}$	$\log_{10}(c_{od,a})$-$\log_{10}(c_{od,b})$
Day1	1448	2435.236	3.1608	3.3865	3.2737	-0.2258
Day2	181	362	2.2577	2.5587	2.4082	-0.3010
Day3	1722.1559	1910.713	3.2361	3.2812	3.2586	-0.0451
Day4	1448	1448	3.1608	3.1608	3.1608	0.0000
Day5	1722.046	1824.366	3.2360	3.2611	3.2486	-0.0251
Day6	2896.2	2048	3.4618	3.3113	3.3866	0.1505
Day7	3444.41	3444.241	3.5371	3.5371	3.5371	0.0000
Day8	1217.618	3444.241	3.0855	3.5371	3.3113	-0.4516
Day9	3444.241	1722.046	3.5371	3.2360	3.3866	0.3010
Day10	1721.972	2896.125	3.2360	3.4618	3.3489	-0.2258

Table S10. Odor concentration measurements of the certified reference materials

Date	CRM used	$c_{od,CRM,a}$ ouE/m^3	$c_{od,CRM,b}$ ouE/m^3	log10($c$od,CRM,a)	log10($c$od,CRM,b)	$\delta_{a,i}$	$\delta_{b,i}$
Day1	n.1	3649.151	3444.311	3.5622	3.5371	0.5622	0.5371
Day2	n.1	2435.49	2896	3.3866	3.4618	0.3866	0.4618
Day3	n.1	4096	4597.246	3.6124	3.6625	0.6124	0.6625
Day4	n.1	5792.5	5792.5	3.7629	3.7629	0.7629	0.7629
Day5	n.1	2047.84	2047.84	3.3113	3.3113	0.3113	0.3113
Day6	n.1	1722.155	2435	3.2361	3.3865	0.2361	0.3865
Day7	n.1	2896.309	3444.311	3.4618	3.5371	0.4618	0.5371
Day8	n.1	2435.49	2047.869	3.3866	3.3113	0.3866	0.3113
Day9	n.1	1722.156	1023.891	3.2361	3.0103	0.2361	0.0103
Day10	n.1	861.078	1217.748	2.9350	3.0856	-0.0650	0.0856

Table S11. Corrected paired odor concentrations of the environmental samples

Date	$\log_{10}(c_{od,a})-\delta_{a,i}$	$\log_{10}(c_{od,b})-\delta_{b,i}$	Corrected difference
Day1	2.5986	2.8494	-0.2509
Day2	1.8711	2.0969	-0.2258
Day3	2.6237	2.6187	0.0050
Day4	2.3979	2.3979	0.0000
Day5	2.9247	2.9498	-0.0251
Day6	3.2258	2.9248	0.3009
Day7	3.0753	3.0000	0.0753
Day8	2.6989	3.2258	-0.5269
Day9	3.3010	3.2258	0.0752
Day10	3.3010	3.3763	-0.0753

3. SUPPLEMENTARY MATERIAL – VOC Species Originating from the Stenter Machine Stacks of Textile Plants

VOC species originating from the stenter stack of textile plants and the average concentrations of these species are shown in Fig. S 3 to Fig. S 10.

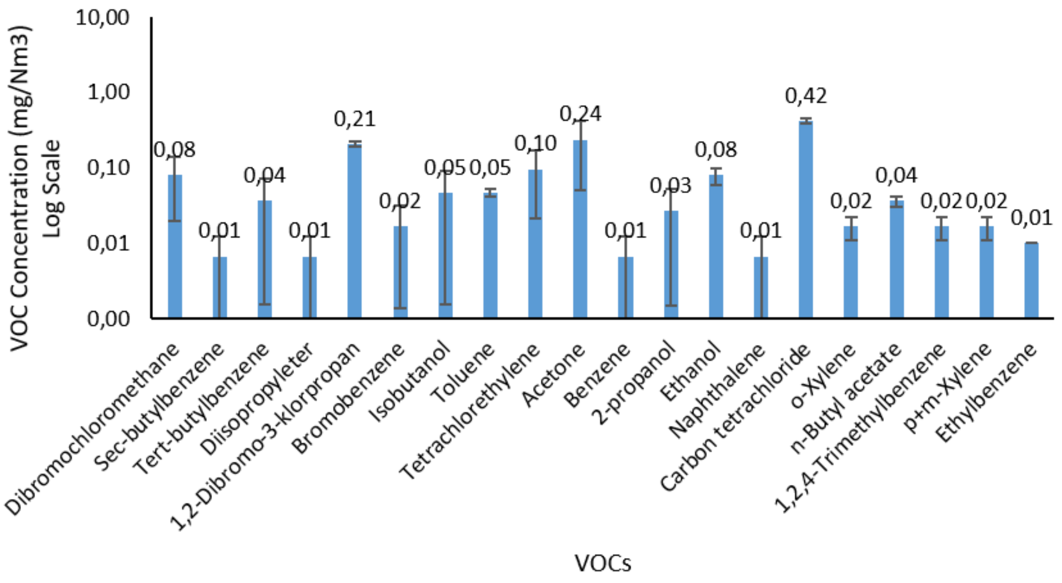

Fig. S 3. The species and concentration levels of VOCs originating from the stenter machine stacks of textile plant 1.

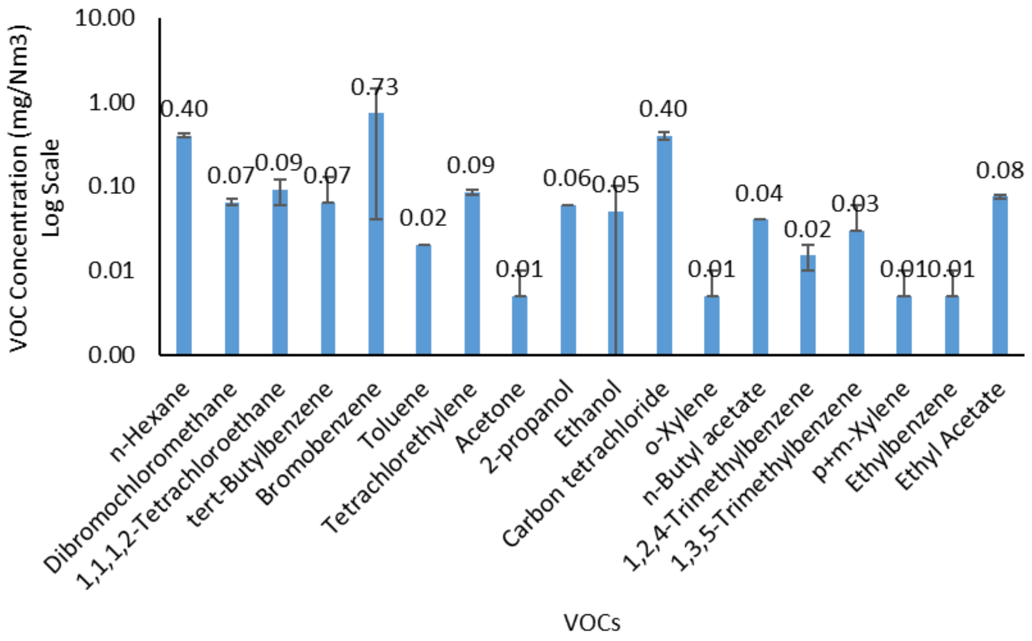

Fig. S 4. The species and concentration levels of VOCs originating from the stenter machine stacks of textile plant 2.

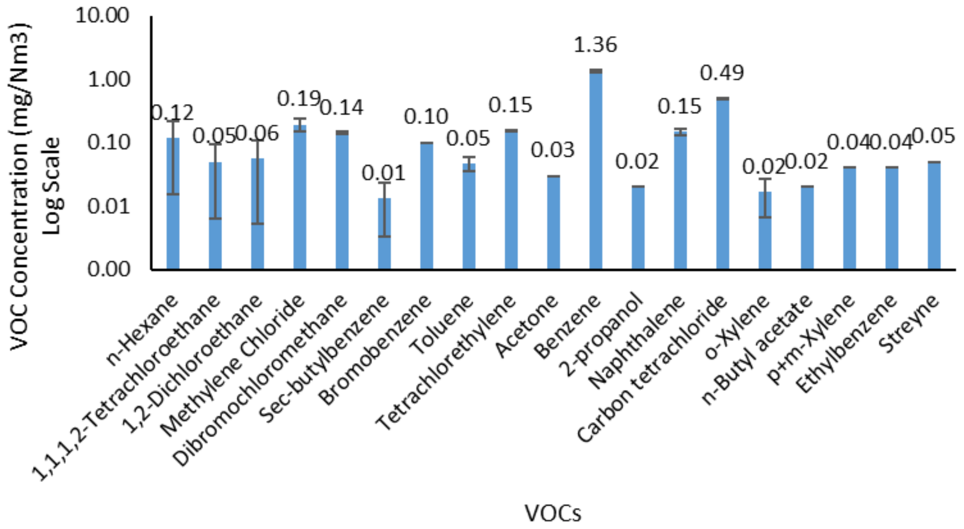

Fig. S 5. The species and concentration levels of VOCs originating from the stenter machine stacks of textile plant 3.

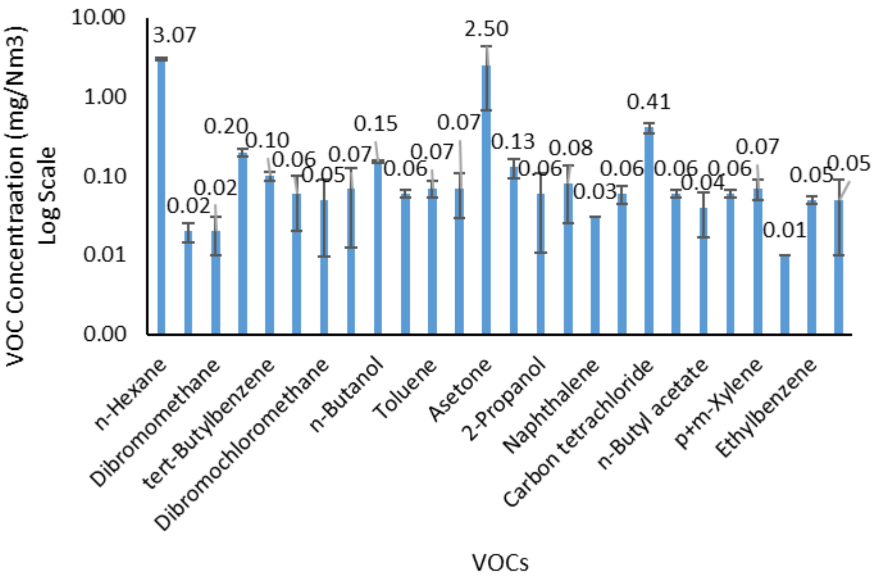

Fig. S 6. The species and concentration levels of VOCs originating from the stenter machine stacks of textile plant 5.

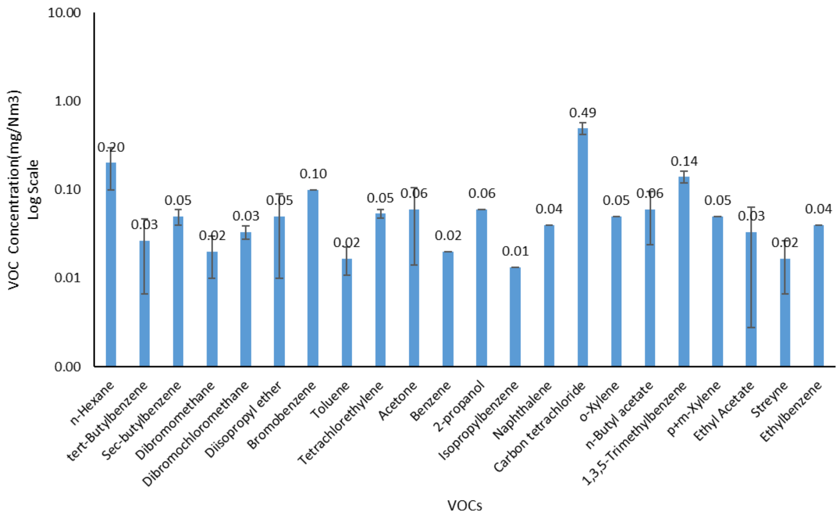

Fig. S 7. The species and concentration levels of VOCs originating from the stenter machine stacks of textile plant 6.

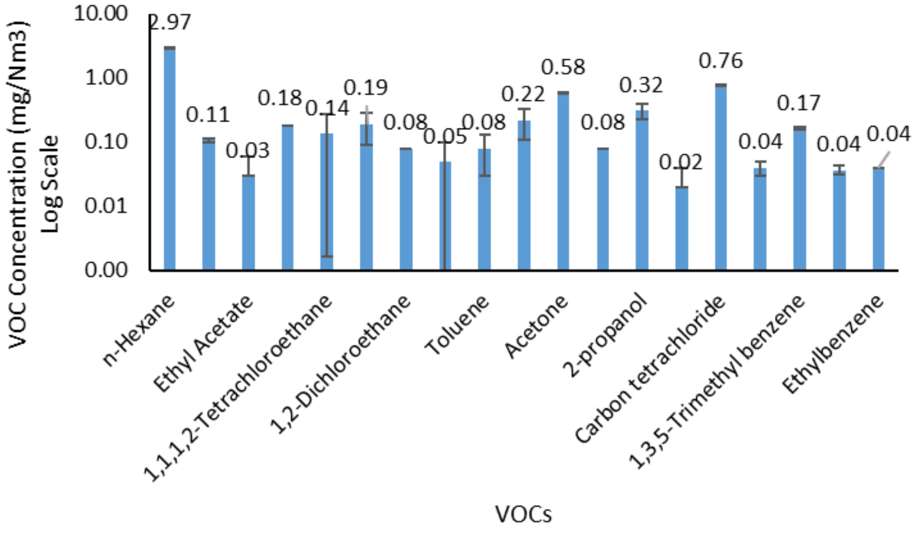

Fig. S 8. The species and concentration levels of VOCs originating from the stenter machine stacks of textile plant 7.

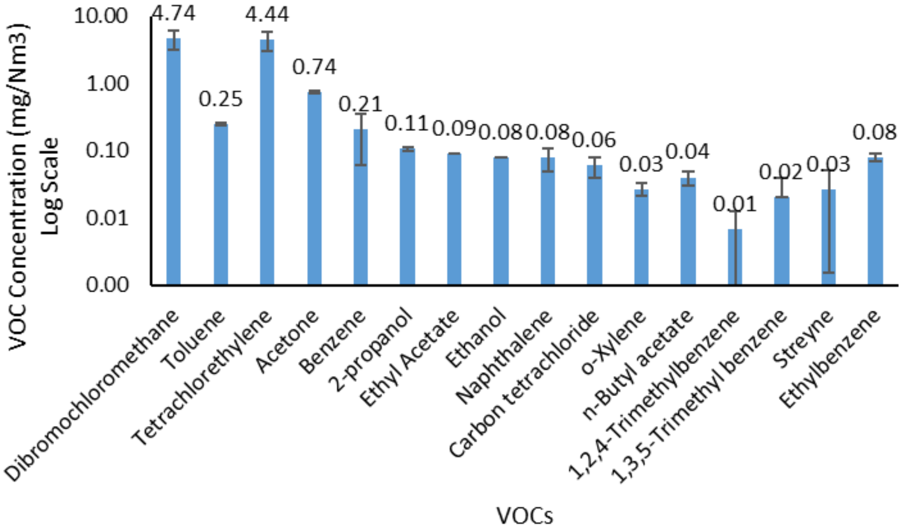

Fig. S 9. The species and concentration levels of VOCs originating from the stenter machine stacks of textile plant 8.

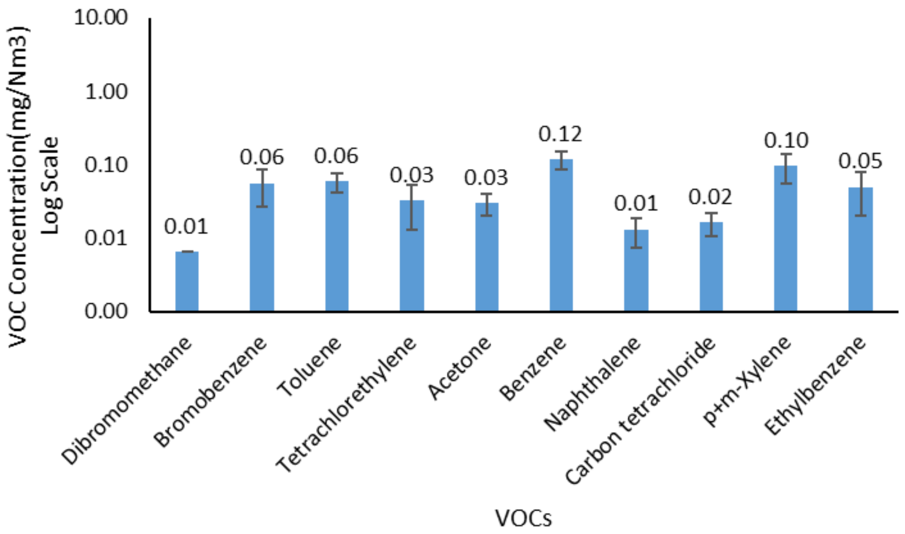

Fig. S 10. The species and concentration levels of VOCs originating from the stenter machine stacks of textile plant 9.

VOC species, number of facilities containing compounds, the minimum, maximum, and average concentrations of these species are shown in Table S 12.

Table S 12. VOC species at textile plants

VOC Species	Number of Textile Plants	Min (mg/Nm³)	Max (mg/Nm³)	Average (mg/Nm³)
1,1,1,2-Tetrachloroethane	4	0.05	0.14	0.08
1,2,4-Trimethylbenzene	4	0.00	0.02	0.01
1,2-Dibromo-3-klorpropan	2	0.20	0.21	0.21
1,2-Dichlorobenzene	1	0.03	0.03	0.03
1,2-Dichloroethane	3	0.06	0.08	0.07
1,3,5-Trimethylbenzene	5	0.02	0.17	0.08
1,3-Dichlorobenzene	1	0.02	0.02	0.02
1,4-Dichlorobenzene	1	0.02	0.02	0.02
2-propanol	7	0.02	0.32	0.09
Acetone	9	0.01	2.5	0.47
Benzene	7	0.01	1.36	0.28
Bromobenzene	7	0.02	0.73	0.16
Carbon tetrachloride	9	0.02	0.76	0.36
Dibromochloromethane	9	0.00	4.74	0.60
Dibromomethane	4	0.00	0.02	0.01
Diisopropyl ether	3	0.01	0.05	0.04
Ethanol	5	0.02	0.08	0.06

Ethyl Acetate	5	0.01	0.09	0.05
Ethylbenzene	9	0.01	0.08	0.04
Isobutanol	2	0.05	0.06	0.06
Isopropylbenzene	1	0.01	0.01	0.01
Methylene Chloride	1	0.19	0.19	0.19
Naphthalene	6	0.01	0.15	0.05
Sec-butylbenzene	3	0.01	0.05	0.02
Styrene	4	0.02	0.06	0.04
Tert-butylbenzene	1	0.04	0.04	0.04
Tetrachlorethylene	9	0.03	4.44	0.59
Toluene	9	0.02	0.25	0.07
n-Butanol	2	0.15	0.18	0.16
n-Butyl acetate	8	0.01	0.06	0.04
n-Heptane	2	0.02	0.11	0.07
n-Hexane	5	0.12	3.07	1.35
o-Xylene	7	0.00	0.06	0.03
p+m-Xylene	9	0.00	0.10	0.04
tert-Butylbenzene	4	0.03	0.10	0.07
